# Clinical Presentations and Outcomes Related to Tuberculosis in Children Younger Than 2 Years of Age in Catalonia

**DOI:** 10.3389/fped.2019.00238

**Published:** 2019-06-11

**Authors:** Antoni Soriano-Arandes, Silvia Brugueras, Alejandro Rodríguez Chitiva, Antoni Noguera-Julian, Àngels Orcau, Andrea Martín-Nalda, Joan P. Millet, Teresa Vallmanya, Maria Méndez, Maite Coll-Sibina, Luis Mayol, Asumpció Clopés, Valentí Pineda, Lourdes García, Nuria López, Olga Calavia, Neus Rius, Tomas M. Pérez-Porcuna, Pere Soler-Palacín, Joan A. Caylà

**Affiliations:** ^1^Pediatric Infectious Diseases and Immunodeficiencies Unit, Hospital Universitari Vall d'Hebron, Barcelona, Spain; ^2^Servei Epidemiologia, Agència de Salut Pública de Barcelona, Barcelona, Spain; ^3^Centre for Biomedical Research in Epidemiology & Public Health (CIBER Epidemiología y Salud Pública - CIBERESP), Barcelona, Spain; ^4^Departament de Pediatria, d'Obstetrícia i Ginecologia i de Medicina Preventiva, Universitat Autònoma de Barcelona, Barcelona, Spain; ^5^Hospital Universitari Sant Joan de Déu, Pediatrics, Esplugues de Llobregat, Spain; ^6^Departament de Pediatria, Universitat de Barcelona, Barcelona, Spain; ^7^Red de Investigación Translacional en Infectología Pediátrica, Madrid, Spain; ^8^Pediatrics, Hospital Universitari Arnau de Vilanova, Lleida, Spain; ^9^Pediatrics, Hospital Universitari Germans Trias i Pujol, Badalona, Spain; ^10^Pediatrics, Hospital General de Granollers, Granollers, Spain; ^11^Pediatrics, Hospital Universitari Josep Trueta, Girona, Spain; ^12^Pediatrics, Hospital Pius, Valls, Spain; ^13^Pediatrics, Hospital Universitari Parc Taulí, Sabadell, Spain; ^14^Pediatrics, Consorci Sanitari del Maresme, Mataró, Spain; ^15^Pediatrics, Hospital Universitari del Mar, Barcelona, Spain; ^16^Pediatrics, Hospital Universitari Joan XXIII, Tarragona, Spain; ^17^Pediatrics, Hospital Universitari Sant Joan de Reus, Reus, Spain; ^18^Unitat clínica de Tuberculosi i Salut Internacional, Fundació Recerca Mútua Terrassa, Servei de Pediatria, Hospital Universitari Mútua de Terrassa, Terrassa, Spain; ^19^Foundation of TB Research Unit of Barcelona, Barcelona, Spain

**Keywords:** tuberculosis, infants, low-incidence country, complications, sequelae, risk factors

## Abstract

**Introduction:** Children younger than 2 years have an increased risk of complications associated with tuberculosis (TB) due to the immaturity of the innate and adaptive immune response. We aimed to identify TB clinical presentations and outcomes as well as risk factors for complications in this age group.

**Materials and Methods:** Multicenter, retrospective, cross-sectional study of TB cases in children aged <2 years in Catalonia (2005–2013). Epidemiological and clinical data were collected from the hospital medical records. TB complications, sequelae included, were defined as any tissue damage generating functional or anatomical impairment after being diagnosed or after TB treatment being completed. Statistical analyses were based on bivariate chi-square and multivariate logistic regression, and it was carried out with Stata® version 13.1. Odds ratios (OR) and its 95% confidence intervals were calculated (CI).

**Results:** A total of 134 patients were included, 50.7% were male, the median [IQR] age was 13[8-18] months, and 18.7% (25/134) showed TB-associated complications. Pulmonary TB was diagnosed in 94.0% (126/134) of children, and the most common complications were lobar collapse (6/126). TB meningitis was diagnosed in 14/134 (10.4%), and hydrocephalus and mental impairment occurred in 1 and 2 patients, respectively. Two patients with spinal TB developed vertebral destruction and paraplegia, respectively. Only one of the patients died. At multivariate level, tachypnea (OR = 4.24; 95% CI 1.17–15.35) and meningeal (OR = 52.21; 95% CI 10.05–271.2) or combined/extrapulmonary forms (OR = 11.3; 95% CI 2.85–45.1) were associated with the development of TB complications.

**Discussion:** TB complications are common in children under 2 years old. Extrapulmonary TB forms in this pediatric age remain a challenge and require prompt diagnosis and treatment in order to prevent them. The presence of tachypnea at the time of TB diagnosis is an independent associated factor to the development of TB complications in infants. This clinical sign should be closely monitored in patients in this age group. It is necessary to perform further studies in this age group in a prospective design in order to understand whether there are other factors associated to TB complications.

## Introduction

An estimated one million children were diagnosed with tuberculosis (TB) in 2016, according to World Health Organization (WHO) data (1). In the European WHO region, the European Centre for Disease Prevention and Control (ECDC) estimated that Spain, the United Kingdom, Romania, and France contributed 62% of pediatric TB cases occurring in the European Union/European Economic Area countries (2).

However, the true global incidence and prevalence of childhood TB remains uncertain due to lack of microbiological confirmation of active TB in the majority of children (3). Children typically have a paucibacillary disease, which hinders the detection of *Mycobacterium tuberculosis* in biological samples. Also, respiratory specimens (i.e., sputum) are difficult to obtain in young children and, consequently most children with active TB worldwide are started on treatment based solely on clinical history, clinical symptoms, and radiological signs (3). Microscopic examination of sputum smears is the key to diagnosis in most countries, but its usefulness is limited in young children with paucibacillary disease who are also unable to expectorate (4).

Children in whom *M. tuberculosis* infection is detected, young children and those with recent exposure are at increased risk for progression to disease (4). Knowledge of the child's status regarding exposure intensity to the index case modifies the pre-test probability of disease and the positive predictive value of subsequent investigations (4). In fact, children under two account for the major part of the elevated morbidity and mortality associated with TB (5). Moreover, the diversity of the clinical presentation in infants and young children and the non-specific nature of most signs and symptoms complicate TB diagnosis at this age. Constitutional symptoms often include failure to thrive and reduced playfulness (4). Non-specific clinical manifestations, particularly in meningeal forms, further complicate early recognition of the disease in these infants (6, 7).

Finally, the recognition of cellular immune response against *M. tuberculosis* is based on two main tests, the tuberculin skin test (TST) and the interferon-γ release assays (IGRAs). However, these tests fail to differentiate *M. tuberculosis* infection from active disease, and their sensitivity is lower in this age group than in older children, making it more difficult to establish diagnosis of infection (4). The contribution of all these factors leads to delay in diagnosis that can be crucial for the patient's clinical outcome and complication occurrence (6, 7).

Literature is scarce on treatment outcome, complications and sequelae occurring in TB diagnosed in children under two. Wiseman et al. (6) described complications as any infiltration or compression of anatomical structures adjacent to the affected site (i.e., neurologic, vascular, bronchial, cardiac, and bone complications), with the potential to cause a functional deficit.

The aims of this study were: 1/ to determine the prevalence rate of TB clinical presentations, outcomes and complications occurring in a large cohort of children with TB under two, and 2/ to investigate the factors associated with these complications. Knowledge of these factors will help in applying preventive measures.

## Materials and Methods

### Design and Setting

This was a multicentric cross-sectional study of pediatric TB cases diagnosed in children under 2 years of age from January 2005 to December 2013 in 10 hospitals which are part of the public Healthcare Network of Catalonia, Spain.

During 2013, the last year of our period of inclusion in the study, a total of 1,135 TB cases were registered in Catalonia, which represents a rate of 15.1 cases per 100,000 population; pediatric patients, aged under 15 years old accounted for 8.5% of the total^1^. In Barcelona, capital city of Catalonia, 1.2% (92 cases) of the total TB registered during the period of 2000-2017 affected children under 2 years of age, and incidence of TB showed a progressive and declining trend from 45 to 11 cases per 100,000 population (Dr. A Orcau, TB Control Program Barcelona—as yet unpublished data).

Data were retrospectively collected from the patient's medical history at the time of TB diagnosis (in hospital or healthcare center). Data related to subsequent clinical evolution at follow-up visits in the Primary Healthcare Centers was collected through the ECAP system (“Estació clínica d'atenció primària”), which is the computerized clinical history used by all professionals in the primary care network of Catalonia, and the shared clinical history of Catalonia. Any health professional working in any of the Catalonian health-care centers can obtain information on patient follow-up regardless of contact with the hospital where the patient was diagnosed or treated for TB. Patients were identified at each center by the local participating physician and monitored at least until the end of TB treatment. They were also monitored until the end of 2015 through data obtained from patients' clinical records. Data were collected at the time of TB diagnosis, at completion of TB treatment again, and at completion of follow-up procedure (December 31, 2015).

### Variables

Demographical, epidemiological, and clinical data was collected. Variables collected for the study were the following:

Demographical: Sex, date of birth, country of birth, and date of arrival to Europe in the case of immigrant children.

Epidemiological: Household with TB cases, Bacillus Calmette-Guérin (BCG) vaccination, contact tracing, and resistance test for the index case.

Clinical: Duration of symptoms prior to diagnosis, poor weight gain, respiratory distress, tachypnea, intensive care admission, fever, hepatomegaly, splenomegaly, poor nutrition, irritability, neurological symptoms, lymph nodes, gastrointestinal symptoms, and vomiting.

Complementary exams: Chest X-ray (CXR), chest computed tomography (CT), human immunodeficiency virus (HIV) test, clinical TB form, microbiological diagnosis, diagnostic tests for microbiological diagnosis, and tuberculin skin test (TST or Mantoux test).

Treatment: type of TB treatment and date commenced, length of therapy (months), and completion of treatment.

Outcome: Complications secondary to TB (yes/no), and site of complications.

### Definitions

- **Case of tuberculosis** was defined as patient meeting following two criteria: (1) Presence of clinical or radiological signs and/or symptoms compatible with TB disease of any location, as long as there is no evidence of another disease to explain their existence. (2) Prescription of standard anti-tuberculosis treatment, generally using three or more drugs. This definition includes confirmed and suspected cases (8, 9).
- **TB complication** was defined as any tissue injury leading to functional or anatomical impairment after completion of TB treatment or after TB diagnosis (7). TB sequelae were also included as a specific type of complication and defined as any anatomic lesion produced and maintained during a long-term period (at least 2 years) after completion of TB treatment. This includes cavitation, focal secondary fibrosis, bronchiectasis, starchy or linear fibrous healing of a cured cavity, destroyed lung on finalizing treatment, airway lesions including bronchiectasis, bronchial stenosis, and even tracheal deformities; In the vascular system: bronchial arteritis, thrombosis, bronchial artery dilatations, aneurysms of the bronchial arteries, pulmonary hypertension compromise secondary to destruction of the pulmonary vascular bed and chronic pulmonary heart; mediastinal lesions, with displacement, mediastinal fibrosis, hilar, and mediastinal lymph node calcifications, esophageal fistulas, chronic pneumothorax, with or without fluid effusion; and lesions of the thoracic walls, with costal retractions or alterations produced by surgical procedures (10). TB meningitis complications were included when any of the following was present: tuberculoma, cavernous sinus syndrome, cranial nerve palsy, stroke, cerebral edema/hydrocephalus, mental impairment/memory loss, spinal involvement, audiologic/visual impairment, epilepsy, and hypothalamic syndrome (11).
- **Low weight gain** was defined as the inability to maintain an adequate weight curve, established by a percentile drop during the period prior to TB diagnosis on standardized age and sex chart (12).
- **Tachypnea** was defined according to WHO (13) as following: respiratory rate (RR) of > 60 per minute in children under 2 months, > 50 per minute in children between 2 and 12 months, and > 40 per minute in children older than 12 months.
- **Household contact:** when the index case lives in the same flat/apartment/house as diagnosed child.
- **TST** was used to screen the subject under study with bacillary antigens, which causes a delayed cellular immune response in the infected individual. We used a dose of 2UT (0.1 ml) of PPD RT 23 with Tween 80. The injection was given on the inside forearm, and the test reading was carried out within 48–72 h. The transverse diameter of the induration produced in the infected children marked the result of this test measured in millimeters.

### Analysis

A descriptive analysis of the variables was carried out. Categorical variables were expressed in number and percentage. Continuous variables were expressed as the median and interquartile range (IQR). The distribution of the main variable was compared with the remaining variables using the Fisher exact test (dichotomous variables) or Mann-Whitney *U*-test (continuous variables). Comparison between patients with and without complications took place. At the bivariate level, associations were examined using the odds ratio (OR) with its 95% confidence interval (CI). The associated variables were analyzed at the multivariate level by logistic regression following the Bayesian multiple imputation methods. Significance was set at a *p*-value of ≤ 0.05. The statistical analysis was performed with the Stata® (version 13.1) software package (College Station, Texas, USA).

### Ethics

Confidentiality of data was maintained at all times in accordance with the guidelines of Organic Law 15/1999 (Ley Orgánica 15/1999), “Protection of Personal Data.” This study was evaluated and approved by the Clinical Research Ethics Committee of the Hospital Universitari Vall d'Hebron (CEIC—HUVH, project PR(AMI)349/2015), as the coordination center. In order to participate each center obtained approval from the corresponding Ethics Committee. As this was a retrospective study that did not involve interventions other than usual clinical practice and in accordance with national legislation informed consent was not required.

## Results

A total of 134 patients was included and follow-up information was collected until the end of 2015. The median duration of follow-up was 86 months (range: 61–106 months). Epidemiological and microbiological characteristics of the study are described in Table 1. It was relevant that almost all of them were Spanish-born, had a positive TST and the CXR or thoracic CT were compatible with TB. Regarding CT, the test was performed in 63 children, of which: 59 were compatible with TB, 2 abnormal but unlikely TB and 2 were normal. All patients except one completed TB treatment, and the BCG vaccination coverage was low. Most of the children had an active TB case at home (household adult TB contact). The cases in which there were other TB cases in the home had more exclusively pulmonary forms than those with no active TB cases in the home (*p* = 0.03). Moreover, those with no TB cases in the home were older (>12 months of age: 73.9 vs. 45.3% *p*-value = 0.002), presented more symptoms before diagnosis (87 vs. 44% *p*-value < 0.001) and suffered more TB related complications (32.6 vs. 10.7% *p*-value = 0.003). Microbiological investigation was performed in 110/134 (82.1%) of the TB cases. Throughout all TB cases included in the study, less than one third were confirmed by microbiology diagnostic tests (44/134), 4/106 (3.8%) by acid-fast bacilli (AFB) strain, 13/47 (27.7%) by GenXpert®MTB/RIF (Cepheid®) and 27/110 (24.5%) by culture. Among those with microbiological study, 44/110 (40%) were confirmed with any of the tests (AFB or GenXpert®MTB/RIF or culture). Two cases of multi-drug resistance TB (MDR-TB) were detected. None of the children tested positive for HIV co-infection. One patient died (case fatality rate of 0.7%) due to neurological complications after TB meningitis.

**Table 1:** Epidemiological and clinical characteristics of 134 children with tuberculosis under 2 years old during the 2005-2013 period in Catalonia.

**Variable**	**Frequency/total TB cases (%)**	**Frequency/only pulmonary TB cases (%)**
Female	66/134 (49.3)	48/101 (47.5)
Age in months (median, IQR)	13 (8–18)	13 (9–18)
Age 0–12 months	56/134 (41.8)	42/101 (41.6)
Spanish born	127/134 (94.8)	94/101 (93.1)
Country of exposition (VFR)	10/134 (7.5)	4/101 (4.0)
BCG vaccination	3/134 (2.2)	3/101 (3.0)
TST positive	122/134 (91.0)	94/101 (93.1)
Chest XR or CT consistent with TB	113/134 (84.3)	89/101 (88.1)
Microbiological confirmation	44/134 (32.8)	27/101 (26.7)
DST of index case (known)	60/134 (44.8)	47/101 (46.5)
Sensitive to first line drugs	58/60 (96.7)	47/47 (100.0)
MDR-TB	2/60 (3.3)	0/47 (0.0)
Contact investigation completed	98/130 (75.4)	79/98 (80.6)
Active TB case at home^*^	75/121 (62.0)	62/92 (67.4)
Treatment completion	132/133 (99.3)	100/100 (100.0)

*TB, tuberculosis; IQR, interquartile range; VFR, visiting friends and relatives; BCG, bacillus Calmette-Guérin vaccine; TST, tuberculin skin test; XR, X-ray; CT, computed tomography; MDR-TB, multi-drug resistant tuberculosis; DST, drug susceptibility testing*.

* *It is statistically significant the association between the presence of active TB case at home and the exclusively pulmonary TB forms (p = 0.03), the younger age (p = 0.002), less symptomatology (p < 0.001), and less TB related complications (p = 0.003) when compared to the cases with no active TB case at home*.

Table 2 shows a description of all TB-related clinical manifestations of study subjects. Over one-third of the children were asymptomatic (37.3%; 50/134) at the moment of TB diagnosis. In pulmonary TB, this percentage was even higher representing nearly half the cases. On the contrary, cases of TB meningitis were almost uniformly symptomatic on TB diagnosis. The most common clinical signs and symptoms on diagnosis were fever >38°C, tachypnea, poor feeding, poor weight gain, and irritability.

**Table 2:** Clinical characteristics at tuberculosis diagnosis in 134 children under 2 years old during the 2005-2013 period in Catalonia according to the clinical form.

**Variable**	**Pulmonary TB (*****N*** **=** **101)**		**TB meningitis (*****N*** **=** **14)**		**Total TB (*****N*** **=** **134)**	
	***N***	**(%)**	***N***	**(%)**	***N***	**(%)**
Clinical manifestations (signs or symptoms)	56	55.4	13	92.9	84	62.7
Symptoms duration <2 weeks	39	38.6	9	64.3	58	43.3
Duration > 2 weeks	17	16.8	4	28.6	26	19.4
Respiratory distress	6	5.9	3	21.4	12	9.0
Tachypnea	30	29.7	6	42.9	41	30.6
Fever > 38°C	39	38.6	9	64.3	56	41.8
Poor feeding	9	8.9	3	21.4	16	11.9
Poor weight gain	7	6.9	3	21.4	13	9.7
Intensive care admission required	0	0.0	6	42.9	6	4.5
Hepatomegaly	3	3.0	1	7.1	6	4.5
Splenomegaly	0	0.0	1	7.1	3	2.2
Irritability	6	5.9	7	41.2	13	9.7
Neurological signs	0	0.0	6	42.9	7	5.2
Lymph nodes palpable	2	2.0	1	7.1	9	6.7
Gastrointestinal symptoms (diarrhea, abdominal distention)	7	6.9	2	14.3	9	6.7
Vomiting	5	5.0	3	21.4	8	6.0

*TB, tuberculosis*.

Pulmonary TB was the most common clinical form, but nearly 20% of the cases (25/126) had other organ or system involvement. The most frequent extrapulmonary TB cases were meningeal, lymphatic, pleural, miliary, osseous, and genitourinary (Table 3).

**Table 3:** Clinical forms of tuberculosis in 134 patients under 2 years old according to lung involvement.

**Clinical presentation**	**Total**	
	***N*^∧^**	**%**
Pulmonary	126	94.0
Meningeal	14	10.4
Lymphatic	13	9.7
Pleural	4	3.0
Miliary	4	3.0
Osseous	2	1.5
Genitourinary	1	0.7
Exclusively Pulmonary	**101**	**75.4**
Combined (Pulmonary and extrapulmonary)	**25**	**18.7**
Exclusively extrapulmonary	**8**	**6.0**

∧ *The same TB case may be in more than one category depending on the affected organs*.

*Bold values include all TB cases categorized according to lung involvement. Values without bold type classify cases based on the affected organ*.

Among the 14 cases with TB meningitis, 12 were investigated using AFB stain, GenXpert®, and culture, and 2 using only AFB stain and culture. Ten (71.4%) of them were microbiologically confirmed.

Concerning TB-associated complications (Table 4), 18.7% of the children had complications, but the highest percentage was among TB meningitis cases followed by other TB cases and pulmonary TB cases. Lung lobar collapse was the most common pulmonary TB-related complication among the pulmonary TB forms but also among all the TB cases. Bronchospasm was considered a TB related complication as the episodes of wheezing were temporarily correlated after TB diagnosis and none of them had experienced any prior bronchiolitis or wheezing. No paradoxical reactions have been reported among the 134 TB cases.

**Table 4:** Tuberculosis complications in 134 children under 2 years old during the 2005–2013 period in Catalonia.

**Variable**	**Frequency/total TB cases (%)**	**Frequency/only pulmonary TB cases (%)**	**Frequency/total TB meningitis cases (%)**
**Development of complications**			
Yes	25/134 (18.7)	7/101 (6.9)	11/14 (78.6)
No	109/134 (81.3)	94/101 (93.1)	3/14 (21.4)
Pulmonary TB-related complications	13/134 (9.7)	7/101 (6.9)	4/14 (28.6)
Bronchiectasis	1/13 (7.7)	0/7 (0.0)	1/4 (25.0)
Lobar collapse	6/13 (46.1)	6/7 (85.7)	3/4 (75.0)
Bronchial stenosis (endobronchitis)	1/13 (7.7)	1/7 (14.3)	0/4 (0.0)
Episodes of bronchospasm	5/13 (38.5)	0/7 (0.0)	0/4 (0.0)
Pleural TB-related complications	2/134 (1.5)		
Pleurisy^*^	2/2 (100.0)		
Lymphatic TB-related complications	3/134 (2.2)		
Ulceration	1/3 (33.3)		
Cellulitis	1/3 (33.3)		
Fistula	1/3 (33.3)		
Meningeal TB-related complications	3/134 (2.2)		3/14 (21.4)
Hydrocephalus/Brain oedema	1/3 (33.3)		1/3 (33.3)
Mental disability/Psychomotor retardation	2/3 (66.7)		2/3 (66.7)
Spinal TB-related complications	2/134 (1.5)		1/14 (7.1)
Abscess/Vertebral destruction	1/2 (50.0)		0/1 (0.0)
Paraplegia/Tetraplegia	1/2 (50.0)		1/1 (100.0)

*TB, tuberculosis*.

* *Pleural TB complications were those requiring surgical intervention secondary to pleural TB as lesions of the thoracic walls with costal retractions or alterations produced by surgical procedures*.

At the bivariate analysis level, to be symptomatic at diagnosis, having extrapulmonary TB, including meningeal and mixed forms, and tachypnea and fever >38°C at diagnosis were associated with the development of TB complications (see Table 5). In contrast, to have a household TB contact was a protective factor for TB complications.

**Table 5:** Factors associated with tuberculosis complications in a cohort of 134 children under 2 years old.

**Variable**	**N complications/total**	***p*-value**	**cOR**	**95% CI**	***p*-value**	**aOR**	**95% CI**
**Gender**							
Male	11/68 (16.2)		1				
Female	14/66 (21.2)	0.45	1.4	0.58–3.34			
**Age**							
0–1	9/56 (16.1)		1				
1–2	16/78 (20.5)	0.51	1.35	0.55–3.32			
**Low weight gain**							
No	20/118 (16.9)		1				
Yes	5/13 (38.5)	0.07	3.06	0.91–10.34			
**Active TB case at home**							
No	15/46 (32.6)		1			1	
Yes	8/75 (10.7)	0.004	0.25	0.09–0.64	0.18	0.44	0.13–1.45
Unknown	2/13 (15.4)	0.24	0.38	0.07–1.91	0.30	0.29	0.03–3.03
**Country of exposition/VFR**							
No	22/124 (17.7)		1				
Yes	3/10 (30.0)	0.35	1.99	0.48–8.29			
**Duration of clinical manifestations**							
0 (No clinical manifestations)	2/50 (4.0)		1				
0–2 weeks	16/58 (27.6)	0.005	9.14	1.98–42.10			
>2 weeks	7/26 (26.9)	0.01	8.84	1.68–46.45			
**TB Localization**							
Pulmonary only	7/101 (6.9)		1			1	
Meningeal	11/14 (78.6)	<0.001	49.24	11.10–218.42	<0.001	52.21	10.05–271.2
Extra or mixt	7/19 (36.8)	0.001	7.83	2.341–26.209	0.001	11.3	2.85–45.1
**Microbiologic confirmation**							
No	13/90 (14.4)		1				
Yes	12/44 (27.3)	0.07	2.22	0.91–5.39			
**Chest XR or CT**							
Done and normal	4/11 (36.4)		1				
Changes compatible with TB	18/113 (15.9)	0.10	0.33	0.09–1.25			
Abnormal but unlikely TB	3/10 (30.0)	0.76	0.75	0.12–4.66			
**Tachypnea**							
No	12/92 (13.0)		1			1	
Yes	13/41 (31.7)	0.01	3.09	1.26–7.57	0.03	4.24	1.17–15.35
**Fever > 38 °C**							
No	9/76 (11.8)		1				
Yes	16/56 (28.6)	0.02	2.98	1.20–7.37			
**Poor feeding**							
No	19/114 (16.7)		1				
Yes	5/16 (31.3)	0.17	2.27	0.71–7.29			

*Bivariate and multivariate analysis. cOR, crude Odds ratio; aOR, adjusted Odds ratio; CI, confidence interval; TB, tuberculosis; VFR, visiting friends and relatives; XR, X-ray; CT, computed tomography*.

In the multivariate analysis, only extrapulmonary and combined clinical TB forms, and the presence of tachypnea at diagnosis remained risk factors for the development of TB complications.

In the case of children suffering pulmonary TB, tachypnea was found to be associated with complications in the bivariate analysis (Table 6).

**Table 6:** Presence of TB complications in a cohort of children under 2 years old with pulmonary TB.

**Variable**	**N complications/total**	***p*-value**	**cOR**	**95% CI**
**Gender**				
Male	3/53 (5.7)		1	
Female	4/48 (8.3)	0.6	1.51	0.32–7.14
**Age**				
0–1	1/42 (2.4)		1	
1–2	6/59 (10.2)	0.16	4.64	0.54–40.08
**Low weight gain**				
No	7/91 (7.7)		1	
Yes	0/7 (0.0)	0.99	0	
**Active TB case at home**				
No	4/30 (13.3)		1	
Yes	3/62 (4.8)	0.17	0.33	0.07–1.58
Unknown	0/9 (0.0)	0.99	0	
**Country of exposition/VFR**				
No	7/97 (7.2)		1	
Yes	0/4 (0.0)	0.99	0	
**Duration of clinical manifestations**				
0 (No clinical manifestations)	1/45 (2.2)		1	
0–2 weeks	5/39 (12.8)	0.09	6.47	0.72–58.00
>2 weeks	1/17 (5.9)	0.48	2.75	0.16–46.61
**Microbiologic confirmation**				
No	7/74 (9.5)		1	
Yes	0/27 (0.0)	0.99	0	
**Chest XR or CT**				
Changes compatible with TB	6/89 (6.7)		1	
Abnormal but unlikely TB	1/7 (14.3)	0.47	2.31	0.24–22.39
Done and normal	0/5 (0.0)	0.99	0	
**Tachypnea**				
No	1/70 (1.4)		1	
Yes	6/30 (20.0)	0.01	17.25	1.97–150.68
**Fever** **>** **38°C**				
No	2/61 (3.3)		1	
Yes	5/39 (12.8)	0.09	4.34	0.80–23.59
**Poor feeding**				
No	7/89 (7.9)		1	
Yes	0/9 (0.0)	0.99	0	

*cOR, crude Odds ratio; CI, confidence interval; TB, tuberculosis; VFR, visiting friends and relatives; XR, X-ray; CT, computed tomography*.

## Discussion

The presence of TB complications in this study carried out in children under two in a low incidence country was relevant, affecting nearly one in five of the cases studied. Risk factors for TB complications at the time of TB diagnosis were extrapulmonary or combined forms and tachypnea.

Although especially younger children are found to have more illnesses, to develop TB easily and also suffer the most severe forms of the disease, there is a lack of information regarding the incidence of TB complications in this age range. In existing scientific databases such as MEDLINE through PubMed® free search engine, most of the articles studying this issue are limited to high-burden TB countries such as South Africa (14, 15), to specific clinical forms such as TB meningitis (16, 17), or to reporting rare complications such as hypercalcemia as an extrapulmonary TB complication (18). We found a study undertaken among 80 children diagnosed with TB at a medical center in southern Taiwan over a period of 24 years (1988–2012) with a similar approach to our study (19). However, this study, published in 2013, was also principally focused on central nervous system (CNS) complications due to TB meningitis (19). It would therefore be correct to say that factors related to TB complications in children under 2 years old in country with low-incidence TB remain a surprisingly under-studied aspect.

The World Health Organization estimated that more than 80% of TB cases in children under 14 are concentrated in 22 developing countries (1), mainly in Africa and South East Asia. The difficulty of diagnosis, lack of resources for contact tracing in many areas and limited pediatric surveillance data in TB control programs (20) are some of the main reasons for lack of knowledge on the real incidence of childhood TB worldwide and subsequently to make an estimate of how many of these children will develop TB complications after treatment.

Regarding the epidemiological characteristics of our sample study, it is important to highlight that most of them were born in Spain (94.8%). They were therefore infected in a low-incidence TB environment; only 7.5% of them were exposed in high-incidence TB countries overseas. The existence of a household TB case was associated with pulmonary TB forms in children when compared to disseminated, meningeal, or combined forms (*p* = 0.03). This could be explained by earlier TB diagnosis when children are included in the contact tracing study with the index case in the same household. Consequently, delay in diagnosis and development into more advanced forms of the disease tends to diminish. Contact tracing investigation was completed in 75.4% of the cases, a higher percentage than that observed in other studies in high-burden countries such as South Africa (14), and similar to that observed in Barcelona (21). Microbiological confirmation was achieved in nearly one-third of the children (32.8%), representing a higher percentage of confirmed TB cases when compared to other studies also from South Africa (15), Mozambique (22), or Ethiopia (23). The percentage of cases with microbiological confirmation was limited, due to the lower performance of microbiological studies in children, especially in younger ones (3), and partly due to the fact that good contact investigations are done which favor early diagnosis and avoid advanced and complicated forms. Finally, the rate of treatment completion was achieved in nearly 100% of our patients which is remarkable data compared to other studies (15, 22).

Regarding clinical manifestations, our study shows great differences as compared to adult TB symptomatology. Fever was absent in most of the recruited children when taking into account total TB cases but was more prevalent in meningeal than in pulmonary cases. Respiratory distress including tachypnea was also more frequent in TB meningitis cases than in pulmonary forms, presumably due to the CNS involvement in disseminated or meningeal TB.

In our study, 18.7% of the TB patients developed TB-associated complications, and one of them died (0.7%). Comparisons with other studies are difficult due to the lack of published data. The highest percentage of TB complications was observed among patients with TB meningitis, followed by the combined forms. A slightly higher rate of TB complications was observed in a study among Spanish children diagnosed with pulmonary TB (24). Lobar collapse was the most frequent complication in children under 2 with pulmonary TB. The same was observed in another study where lung collapse might occur secondary to an endobronchial obstruction or an extrinsic compression caused by lymphadenopathy (24). In our study, bronchiectasis was found in only one case; this was likely to have been caused by fibrosis of the bronchial wall occurring during disease or by traction resulting from areas of scar tissue (10). Regarding bronchospasm, it was possible to temporarily correlate the episodes of wheezing after TB diagnosis. However, we were not able to demonstrate whether these episodes were caused by TB.

The incidence of the pleural involvement varies considerably, being much higher in high-incidence TB areas, affecting up to 40% of cases in South Africa (25). In Canada (26), a country with a lower TB incidence than Spain, Pineda et al. (27) detected pleural involvement in 4% of 202 patients with pulmonary TB, whereas in our study, only 1.5% of children showed pleural TB related complications.

Peripheral lymphadenopathy is the most common clinical sign in infants diagnosed with TB, seen in 25–35% of extrathoracic TB cases, according to daita from the Spanish Society of Pediatric Infectious Diseases (28). In our study, 10 children (7.5%) had extrapulmonary TB complications—three of them were lymphatic TB-related (cellulitis, fistula, and ulceration)—accounting for 40% of all TB-complications. The explanation for this could be due to the greater risk of disseminated and combined forms in infants when compared to older children or adults.

TB meningitis is the most severe and life-threatening form of the disease in infants (29). Prompt diagnosis and treatment is essential to the improvement in prognosis of the disease by decreasing the case fatality rate (CFR) and the incidence of irreversible complications (30). The main risk factors for an unfavorable prognosis, including death, are a low Glasgow Coma Scale value at diagnosis, and age under 2 (30). Ten percent of our children had TB meningitis, much lower than in the under 3-year-old age-group of the largest cohort of culture-confirmed childhood TB from South Africa (14). In a study on TB meningitis in Romania, complications affected 36 and 14% of children and adults, respectively (31); illustrating the greater risk in children with this condition. Otherwise, we found TB-complications in 78.6% of the cases of TB meningitis in our study, among which 21.4% (3/14) were related to CNS involvement, including psychomotor retardation and hydrocephalus.

Lastly, musculoskeletal involvement has been described in 10–20% of extrapulmonary TB cases, which would account for 1–2% of all TB cases (32), data that is very similar to our results (25 and 1.5%, respectively).

Among all TB cases, at bivariate analysis, we found that the presence of fever or tachypnea and the extrapulmonary or combined forms were associated with TB complications. Otherwise, as mentioned above, having a household TB contact was a protective factor of these complications. At the multivariate level, tachypnea and extrapulmonary or combined forms were associated factors with TB complications. Nevertheless, when the analysis was restricted to pulmonary TB cases, only tachypnea was an associated factor in TB complications.

The fact that tachypnea at the time of TB diagnosis was an independent variable associated to TB complications, could be explained by the fact that it is a compensatory response secondary to hypoxemia for the small lung volume of restrictive lung disease or metabolic acid mechanism of sis caused by advanced TB disease. It is the earliest detectable clinical sign. Compensatory mechanisms, such as tachypnea, also operate to maximize gaseous exchange in diseased lungs (33). Moreover, tachypnea is a useful sign for the diagnosis of childhood pneumonia. It is more specific and reproducible than auscultatory signs (34) and has been identified by the last British clinical updates for the management of community-acquired pneumonia in children as a sign of moderate or severe pneumonia in the risk assessment algorithm (35). However, as a prognosis factor, tachypnea has only been associated to worst outcome in adults with community-acquired pneumonia (36), but not yet in children.

In contrast to the data from studies in areas with high incidence of TB and HIV coinfection such as South Africa (CFR 21%) (37) or Myanmar (CFR 11.4%) (38), there was only one death in our study representing a CFR of 0.7% over the total TB cases. Other studies in Kenya (39) or among MDR TB cases in children (40) showed CFR percentages of 4 and 21%, respectively. Since this is a cross-sectional retrospective study, the data collection process was subject to limitations, and the possibility of missing information exists. It was difficult to obtain long-term data since most of the patients were discharged from the specialized medical unit at completion of treatment. Hence, we cannot ensure that additional complications of the disease did not develop at a later stage. The relatively high percentage of TB meningitis (10.4%) in our sample might not be representative of the childhood TB population, leading to selection bias due to the hospital-based design of our study. However, this difference was minimal if we take into consideration the data provided by the TB Control Program of Barcelona / Public Health Agency of Barcelona for this age-group of children with TB meningitis during the 2000–2017 period (6.5%). Another limitation is that we were able to determine if the children had complications during and after TB treatment, but not when each of the events occurred or how long it lasted. It is not possible to distinguish between TB complications that lasted until the end of TB treatment and long-lasting sequelae (maintained during at least 2 years after TB treatment completion).

In conclusion, our findings show that TB incidence in children under 2-years of age is still relevant in developed countries, and TB complications are frequent. Extrapulmonary TB forms in this pediatric age remain a challenge and require prompt diagnosis and treatment to prevent adverse outcomes. The presence of tachypnea at the time of TB diagnosis is an independent associated factor to the development of TB complications in infants. Hence, this clinical sign should be closely monitored in patients within this age group. It is necessary to perform further studies in this group of children in a prospective study designed to ascertain whether other factors associated with TB complications exist.

## Author Contributions

AS-A: conceptualized and designed the study. AS-A, AR, AN-J, AM-N, TV, MM, MC-S, LM, AC, VP, LG, NL, OC, NR, and PS-P: data collection. AS-A, SB, ÀO, JM, and JC: statistical analysis. AS-A, AR, PS-P, TP-P, SB, ÀO, JM, and JC: drafted initial manuscript. All authors reviewed and revised manuscript, approved final manuscript as submitted.

### Conflict of Interest Statement

The authors declare that the research was conducted in the absence of any commercial or financial relationships that could be construed as a potential conflict of interest.

## Footnotes

^1^http://salutpublica.gencat.cat/ca/detalls/Article/Publicacio-informe-TBC-2015 (accessed September 13, 2018).
